# Single, but not mixed dietary fibers suppress body weight gain and adiposity in high fat-fed mice

**DOI:** 10.3389/fmicb.2025.1544433

**Published:** 2025-02-12

**Authors:** Swang M. Shallangwa, Alexander W. Ross, Peter J. Morgan

**Affiliations:** Rowett Institute, University of Aberdeen, Aberdeen, United Kingdom

**Keywords:** dietary fibers, gut microbiota, obesity, gene expression, gut hormones

## Abstract

Dietary fiber can suppress excess adipose tissue and weight gain in rodents and humans when fed high fat diets. The gut microbiome is thought to have a key role, although exactly how remains unclear. In a tightly controlled murine study, we explored how different types of dietary fiber and doses affect the gut microbiota and gut epithelial gene expression. We show that 10% pectin and 10% FOS suppress high fat diet (HFD)-induced weight gain, effects not seen at 2% doses. Furthermore, 2 and 10% mixtures of dietary fiber were also without effect. Each fiber treatment stimulated a distinct gut microbiota profile at the family and operational taxonomic unit (OTU) level. Mechanistically it is likely that the single 10% fiber dose shifted selected bacteria above some threshold abundance, required to suppress body weight, which was not achieved by the 10% Mix, composed of 4 fibers each at 2.5%. Plasma levels of the gut hormone PYY were elevated by 10% pectin and FOS, but not 10% mixed fibers, and similarly RNA seq revealed some distinct effects of the 10% single fibers on gut epithelial gene expression. These data show how the ability of dietary fiber to suppress HFD-induced weight gain is dependent upon both fiber type and dose. It also shows that the microbial response to dietary fiber is distinct and that there is not a single microbial response associated with the inhibition of adiposity and weight gain. PYY seems key to the latter response, although the role of other factors such as Reg3γ and CCK needs to be explored.

## 1 Introduction

Dietary fiber suppresses food intake and body weight gain in rodents fed a high fat diet and even limits weight gain and adiposity when fed non-obesogenic diets (Slavin and Green, 2007; Shallangwa et al., 2024; Adam et al., 2014; Anastasovska et al., 2012; Cani et al., 2006; Cani et al., 2004; Rodriguez and Delzenne, 2021). Dietary fiber thereby can act as a natural restraint on excess energy intake and obesity. While these effects are often observed there are also reports where dietary fiber either has a weak or no effect on body weight gain (Knapp et al., 2013; Kuo et al., 2013; Shallangwa et al., 2024). In humans, dietary fiber has also been reported to suppress energy intake (Wanders et al., 2011; Slavin, 2013; John et al., 2018; Rasaei et al., 2024), although the effects are more variable and less robust than those seen in rodents. To understand the basis of this inconsistency in response a clearer understanding of the mechanisms through which dietary fiber inhibits food intake and suppresses weight gain is required.

Dietary fiber is a term covering a range of carbohydrates that evade digestion in the upper gut, but which can be fermented into short chain fatty acids by the bacteria resident in the lower gut. Dietary fibers range from insoluble, poorly fermented molecules such as cellulose through to soluble forms, such as pectin and fructooligosaccharide (FOS), which are good substrates for bacterial fermentation. Pectin is naturally found in citrus fruits and apples and is a highly polymerized polysaccharide composed of D-galacturonic acid units. FOS is also present in fruit and vegetables and is a polymer of fructose units. Inulin is also a polymer of fructose units, which is naturally found in a variety of plants. It differs from FOS in terms of chain length with FOS being a linear chain of 2–10 fructose units, whereas inulin has a more crosslinked structure composed of between 10 and 50 fructose units. Beta-glucan is a highly polymerized glucose polysaccharide found in cereals and bran. These differences in sugar backbone and degree of polymerization markedly affect their physico-chemical properties and susceptibility to fermentation by the different gut bacteria.

One of the favored mechanisms of food intake suppression by dietary fiber is through SCFA-mediated stimulation of anorexigenic gut hormone production and release. SCFAs are natural ligands for the G-protein coupled receptors, free fatty acid receptors 2 and 3 (FFAR2 and FFAR3), which are expressed on L-cells in the gut (Tazoe et al., 2009; Karaki et al., 2008; Karaki et al., 2006; Nohr et al., 2013). L-cells are enteroendocrine cells which produce the peptide hormones PYY and GLP-1, both of which are potent inhibitors of food intake in animals and humans (Spreckley and Murphy, 2015). This provides a plausible linkage between microbial fermentation and gut hormone-mediated food intake restraint (Brooks et al., 2017; Tolhurst et al., 2012; Larraufie et al., 2018; Psichas et al., 2015). At present however, the evidence to support this as the primary functional mechanism explaining inhibition of excess energy intake by dietary fiber is lacking. One of the issues is that elevated levels of PYY and GLP-1 have been observed in animals fed dietary fiber without any associated restraint in food intake (Shallangwa et al., 2024). Conversely, there are reports of energy restraint in mice fed dietary fiber, but without associated changes in gut hormones (Anastasovska et al., 2012; Frost et al., 2014).

For this reason, other potential mechanisms have been explored to explain the food intake inhibitory effects of dietary fiber. One of these includes the direct effects of the SCFA, acetate, which is the most abundant microbial fermentation product of dietary fiber metabolism (Anastasovska et al., 2012; Frost et al., 2014). Significantly acetate crosses the gut epithelial layer and enters the circulation and can reach the brain (Frost et al., 2014). There it has been shown, using manganese-enhanced MRI (MEMRI) and CT PET, that acetate activates neurons in the arcuate, ventromedial and paraventricular regions of the hypothalamus, areas known to be involved in the control of food intake (Anastasovska et al., 2012, Frost et al., 2014).

The link between dietary fiber and restrained food intake and body weight gain starts with the gut bacteria, which can ferment the complex polysaccharides. The ability of gut bacteria to ferment specific dietary fibers depends upon the expression of appropriate enzymes in specific bacteria. As a result, some bacteria can digest specific dietary fibers more than others (Chung et al., 2016). From this it can be predicted that different fibers will stimulate distinct microbiota profiles, giving clues to which gut microbiota are most critical to the effects of a specific dietary fiber on energy homeostasis. While the evidence linking specific bacterial profiles or signatures to obesity appears weak (Walker and Hoyles 2023), meta-analysis of several randomized control trials does suggest that certain probiotic species of *Bifidobacterium* or *Lactobacillus* species may significantly reduce body weight in humans (Koutnikova et al., 2019; Suzumura et al., 2019; Wang et al., 2019). While the effects are generally modest, they are nonetheless indicative of microbial efficacy in terms of weight loss. Other bacterial species that have been associated with reduced adiposity and body weight are *Akkermansia muciniphila* and *Christensenella minuta* (Depommier et al., 2019; Everard et al., 2013; Goodrich et al., 2014; Peters et al., 2018; Oki et al., 2016), although clinical validation of their efficacy is still lacking (Dalby, 2023). In a previous study, we demonstrated in non-obese rats that the ability of dietary fibers to suppress food intake and limit body weight gain and adiposity was associated with the presence and absence of a specific gut bacterium, *Allobaculum fili* (Shallangwa et al., 2024). In turn, the responsiveness also seemed to be dependent on the gut microbiota profile of the animals prior to the start of the study (Shallangwa et al., 2024).

This study sought to explore the relationship between the microbiota profile and the body weight/adiposity response in mice fed a high fat diet. We explored the effect of different single fibers (pectin and FOS) as well as combined mixed fibers (pectin, FOS, ß-glucan and inulin) at different doses on the gut microbial profile and the host response in terms of the gene expression profile of the gut epithelium. A key aspect of the experimental design was to limit the within-experimental variation in gut microbiota of the mice at the start of the experiment, as far as practicable, so that any differences in effects of the different fibers on the gut microbiota could be revealed.

## 2 Materials and methods

### 2.1 Diets

Diet recipes are shown in Supplementary Table S1.

Dietary fibers used: Apple pectin: Cat no. 93854-1KG; Merck Life Science United Kingdom Limited, The Old Brickyard, New Road, Dorset SP8 4XT, UK. Fructooligosaccharide, (FOS), Orafti®P95 and FOS + inulin: Synergy 1 generously provided by BENEO GmbH, Maximilianstr. 10, 68,165 Mannheim, Germany. Oat beta-glucan: Cat no. NIGECER000241; Nutraceuticals Group, The Old Smithy, 7 High Street, Merstham, Surrey, RH1 3BA, United Kingdom.

### 2.2 Experimental animals, study design and sample collection

Animal experiments were conducted in line with UK Home Office Animal (Scientific Procedures) Act 1986, conforming to Institutional and national guidelines for the care and use of animals, and with approval by the local ethical review board (AWERB) at the University of Aberdeen. The study was carried out at the Medical Research Facility (MRF) building located at the University of Aberdeen, and under UK Home Office project license number P5ACD03D2 with local study plan number 141221AR.

C57Bl/6 J mice were purchased from Charles River Laboratories UK at 4 weeks of age and from the same breeding unit. They were held in paired housing for 6 weeks before starting the experiment within the Medical Research Facility at the University of Aberdeen and were fed a normal chow diet *ad libitum*, with free access to water.

#### 2.2.1 Study design

**Figure 1:**
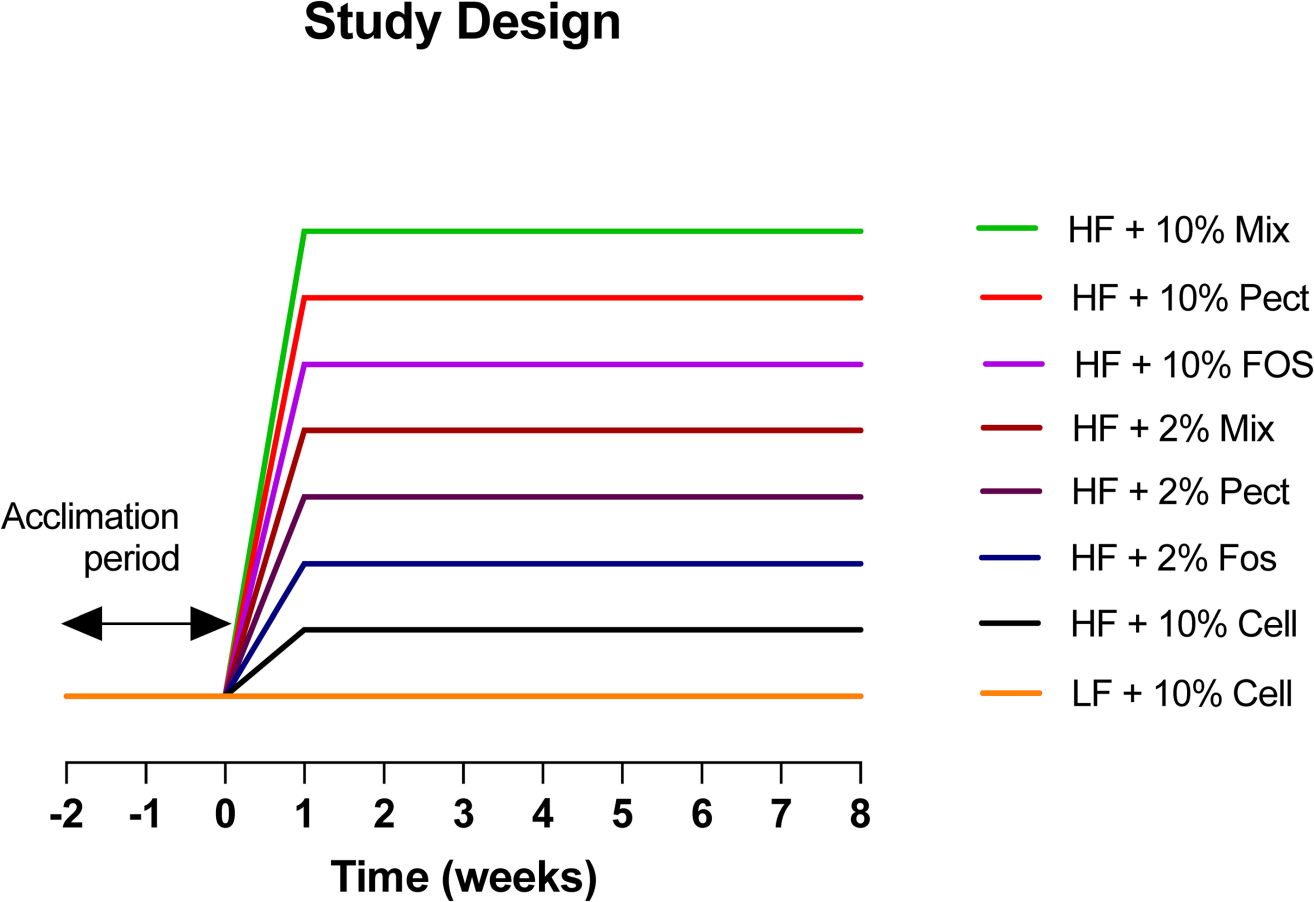
Study design. Eighty C57BL/6J mice were brought in at 4 weeks of age and were pair-housed for 6 weeks prior to being placed onto a LF + 10% cellulose diet for a further two-week acclimation period. The mice were then set up in 8 groups of 10 and assigned to one of the following treatments. (1) HF + 10% Mixed fiber (HF + 10% Mix); (2) HF + 10% Pectin (HF + 10% Pect); (3) HF+ 10% FOS (HF + 10% FOS); (4) HF + 2% Mixed fiber/8% cellulose (HF + 2% Mix); (5) HF + 2% Pectin/8% cellulose (HF + 2% Pect); (6) HF + 2% Fos/8% cellulose (HF + 2% FOS); (7) HF + 10% cellulose (HF + 10% Cell) and (8) LF + 10% cellulose (LF + 10% Cell). Dietary treatments were continued for 8 weeks. FOS = fructooligosaccharide.

The study was set up to test the effects of 2 different types of soluble dietary fiber (FOS and Pectin) at two different doses (2 and 10%) for their ability to restrain body weight gain in C57BL/6 J mice when added to a high fat refined diet. The effects of a mixture of 4 fibers, including FOS, pectin, inulin and beta glucan at the two doses (2 and 10%) were also tested for their effects for comparison against individual fibers. Following the pre-experimental period, mice were randomly weight matched as pairs into 8 groups of 10 mice using the rand feature in Excel, and continued in paired housing. They were fed a low fat (LF) refined +10% cellulose diet (LF + 10% Cell) during a 2-week acclimation period, after which groups of 10 mice were randomly assigned to one of 8 treatments (see Figure 1). One group of mice was maintained on the LF + 10% Cell diet for the 8-week intervention period while the remaining groups of mice were transferred onto one of the following diet treatments: High fat (HF) refined 10% cellulose diet (HF + 10% Cell) or High Fat refined diets where the cellulose was replaced with either high (10%) or low (2%) levels of pectin (HF + Pect), or Fructo-oligosaccharide (FOS) (HF + FOS), or Mixed Fiber (HF + Mix). The 10% Mixed fiber diet contained 2.5% pectin, 2.5% FOS, 2.5% inulin and 2.5% beta glucan and for the 2% Mixed diets, they contained 0.5% pectin, 0.5% FOS, 0.5% inulin and 0.5% beta glucan and cellulose was added at 8%, thereby maintaining comparability to the single fiber diets. The weight restraining effects of the fiber treatments were measured over an 8-week intervention period. Controls were a LF refined diet +10% cellulose and HF refined diet +10% cellulose (see Figure 1 for further details).

#### 2.2.2 Sample collection

Throughout the experimental period, body weights were recorded weekly. Fat mass was measured by Echo MRI scanning at the start of the experimental period and by excision and weighing of the epididymal and retroperitoneal fat depots at the termination of the experiment, due to problems with the Echo MRI machine. Blood samples were collected by cardiac puncture into K_2_EDTA-coated tubes and immediately chilled on ice, then plasma prepared and stored frozen. Gut tissues (colon and ceca) were harvested and weighed. Gut epithelial cells were prepared by scraping the PBS-flushed luminal walls of the colon and saved in tubes containing RNAlater® (Merck cat no. R0901, Merck Life Science United Kingdom Limited, Gillingham, Dorset, United Kingdom) and prepared according to the manufacturer’s instructions and stored at-70°C prior to RNA extraction. Cecal, colon and fecal material (from excreted pellets) was collected and stored at-70°C.

### 2.3 Plasma analysis for PYY and GLP-1

The plasma hormones GLP-1 (total) and PYY were analyzed using a Milliplex® Mouse Metabolic Hormone Expanded Panel kit number MMHE-44 K, 96-Well Plate Assay, following the manufacturer’s instructions and using a BioRad Bioplex 200 instrument (Bio-Rad Laboratories Ltd. The Junction, Station Road, Watford, Hertfordshire, WD17 1ET, United Kingdom).

### 2.4 Analysis of gut microbiota fermentation products

SCFA levels in fecal samples were measured by capillary gas chromatography using the technique developed by Richardson et al. with helium used as the carrier gas (Richardson et al., 1989). Samples were diluted in distilled water and 2-ethylbutyric acid (5 mmol/L) was added as internal standard. The extraction of samples was carried out in diethyl ether and derivatized with N-tert-butyldimethylsilyl-N-methyltrifluoroacetamide. Separation and quantification were performed using Agilent GC HP-1 capillary columns.

### 2.5 DNA extraction

The cecal contents were collected in 2 mL Eppendorf tubes and stored at -20°C. Microbial DNA was extracted from the cecal contents after 2 weeks of storage using FastDNA® SPIN Kit for feces (MP Biomedicals 116,570,200, MP Biomedicals SARL, ILLKirch, France) following manufacturer’s instructions.

### 2.6 PCR amplification of 16S rRNA genes and Illumina MiSeq sequencing

After quality checks of the extracted bacterial DNA using agarose gel visualization, the V1-V2 variable regions of the bacterial 16S rRNA gene were amplified using forward (F) primer MiSeq-27F (5′-AATGATACGGCGACCACCGAGATCTACACTATGGTAATTCCAGMGTTYGATYMTGGCTCAG-3′) and MiSeq-338R (5′-CAAGCAGAAGACGGCATACGAGAT-barcode-AGTCAGTCAGAAGCTGCCTCCCGTAGGAGT-3′) which includes adaptors used for downstream Illumina sequencing. The reverse (R) primer also includes a unique 12-base pair barcode which is important in identifying each sample amplicon (Anastasovska et al., 2012; Frost et al., 2014).

The extracted DNA templates were amplified by PCR using the New England Biolabs Q5® High-Fidelity DNA polymerase (Hertfordshire, United Kingdom). Four separate 25 μL PCR reaction mixtures were prepared for each extracted DNA sample made up of 5 x Q5 buffer (5 μL), 10 mM dNTPs (0.5 μL), 10 μM F primer (1.25 μL), 10 μm R primer (1.25 μL), the template DNA (1 μL, ave. 65 mg/μL), Q5 High-fidelity DNA polymerase (0.25 μL) and Nuclease-Free water (15.75 μL). The conditions set for the PCR were 2 min at 98°C, then 20 cycles of 30 s at 98°C, 30 s at 50°C, 90 s at 72°C; then a final 5-min extension at 72°C which was then followed by a holding temperature of 4°C. After verification of amplified DNA products using agarose gel electrophoresis, the 25 μL of each of the samples were pooled into 1.5 mL sterile microcentrifuge tubes and precipitated with ethanol. Following resuspension, the amplicons were quantified using a Qubit dsDNA HS Assay kit (Invitrogen, CA, United States Q32854). The equimolar mix needed for Illumina MiSeq sequencing was prepared using equal quantities from each of the PCR amplified samples. The amplicons were then sequenced using a MiSeq machine by Center for Genome Enabled Biology and Medicine (CGEBM) at the University of Aberdeen.

### 2.7 Statistical analysis and bioinformatics

The raw sequence data (FASTQ files) obtained from CGEBM of the University of Aberdeen were analyzed using Mothur software package (Schloss et al., 2009) based primarily on the procedure as described by Mothur MiSeq standard operating procedure (Kozich et al., 2013). To start the process, the forward and reverse reads from each of the samples were assembled into pair contigs resulting in a total of 8,654,506 sequences. To improve the quality of the reads, a quality control measure was introduced which screens and removes any paired contigs that are shorter than 280 base pairs and more than 470 base pairs, that had ambiguous bases or included homopolymeric base stretches of 8. Next, SILVA reference database was used to align and map unique sequences, and to mitigate against potential sequencing errors the Pre-cluster, which allows 3 base differences, was run (Huse et al., 2010). A further quality control measure known as UCHIME was employed to detect and remove all chimeric molecules that might have been formed during the PCR amplification process (Edgar et al., 2011). The Ribosomal Database Project (RDP) (release 10) (Wang et al., 2007) was then used as a reference database to assign taxonomic classification for each read. The rest of the reads were then clustered to form operational taxonomic units (OTUs) created at 97% similarity using Mothur. Subsampling was done at 4414 reads per sample to ensure a level playing field for comparison. A statistical method known as Metastats (White et al., 2009), which incorporates Fisher’s exact test was used to make paired comparisons to find out whether there are any OTUs (or higher taxa) that are significantly differentiated between groups. Focus was made on OTUs that had proportional abundance of greater than or equal to 0.5% and the *p* values generated by Metastats were corrected using the Benjamini Hochberg method (Benjamini and Hochberg, 1995) to mitigate against false discovery rate (FDR).

Alpha diversity (diversity within each sample) was determined using observed richness (sobs), estimated total richness (Chao), and Good’s coverage (Finotello et al., 2018) with Mothur software. Principle coordinate analysis (PCoA) plots were created from the Bray Curtis index calculator by generating a distant matrix based on the shared file. Visualization of the mappings of the different groups on the PCoA plots was carried out using R package ggplot2 (Wickham, 2008) revealing the beta diversity of the different samples. Nonparametric analysis of molecular variance (AMOVA) was used to test for significant differences in clustering based on treatment.

### 2.8 RNA seq

Total RNA was extracted from colon mucosal scrapings using RNeasy Mini Kit (QAIGEN, Crawley, United Kingdom) and following the manufacturer’s protocol. Briefly, weighed samples were homogenized in a Precellys homogenizer (6,500 rpm) (Bertin Technologies, Ann Arbor, United States) for 15 s using ~500 mg Zirconia beads (Thistle Scientific, Glasgow, United Kingdom) in a 2.0 mL tube with 400 μL RLT buffer containing *β*-mercaptoethanol. The homogenates were centrifuged in RNeasy spin columns and on-column DNase digestion was carried out before eluting the RNA. Thereafter the extraction process followed the manufacturer’s instructions. A spectrometer (NanoDropR ND-1000 UV, ThermoFisher, United Kingdom) was used to quantify the total RNA, and purity was assessed by measuring the 260/280 nm absorbance ratio and quality was assessed using the 4,200 TapeStation system (Software 3.2, Agilent Technologies Inc., Germany).

Unique dual indexed Illumina libraries were prepared from 500 ng total RNA using the Stranded TruSeq mRNAseq kit according to the manufacturer’s instructions (Illumina, CA). Library molarity was determined by qPCR with SYBR green (Kapa Library Quantification Complete Universal, Roche, CH) on the QuantStudio 6 Flex (ThermoFisher Scientific, United Kingdom) with library size determined on the TapeStation 4,200 (Agilent, CA). Libraries were diluted in 10 mM Tris–HCl, pH 8.5 and equimolar pooled before sequencing and base calling on an Illumina NextSeq500 with v2.5 chemistry and 75 bp single reads and average 35.5 M reads per sample.

Following sequencing, quality control analysis was performed on the sequence reads using FastQC [v.0.11.9] (Andrews, 2016) and the quality reports combined with MultiQC [v1.1] (Ewels et al., 2016). Quality trimming was carried out using cutadapt [v.4.1] (Martin, 2011) and trim-galore [v.0.6.6] (Krueger, 2015) with a quality threshold of Q30. This resulted in a reduction of ~500,000 reads per sample, with >20 million reads per sample remaining.

Trimmed reads were aligned to the GRCm39 reference genome (Church et al., 2011) using hisat2 [v.2.2.0] (Kim et al., 2019), followed by the aligned reads being sorted and filtered using samtools [v.1.14] (Danecek et al., 2021). Aligned read counts were subsequently assigned to genes using the associated annotation file for GRCm39 and the featureCounts program of the subread package [v.2.0.2] (Liao et al., 2014).

## 3 Results

### 3.1 Effects of fiber on body weight

**Figure 2:**
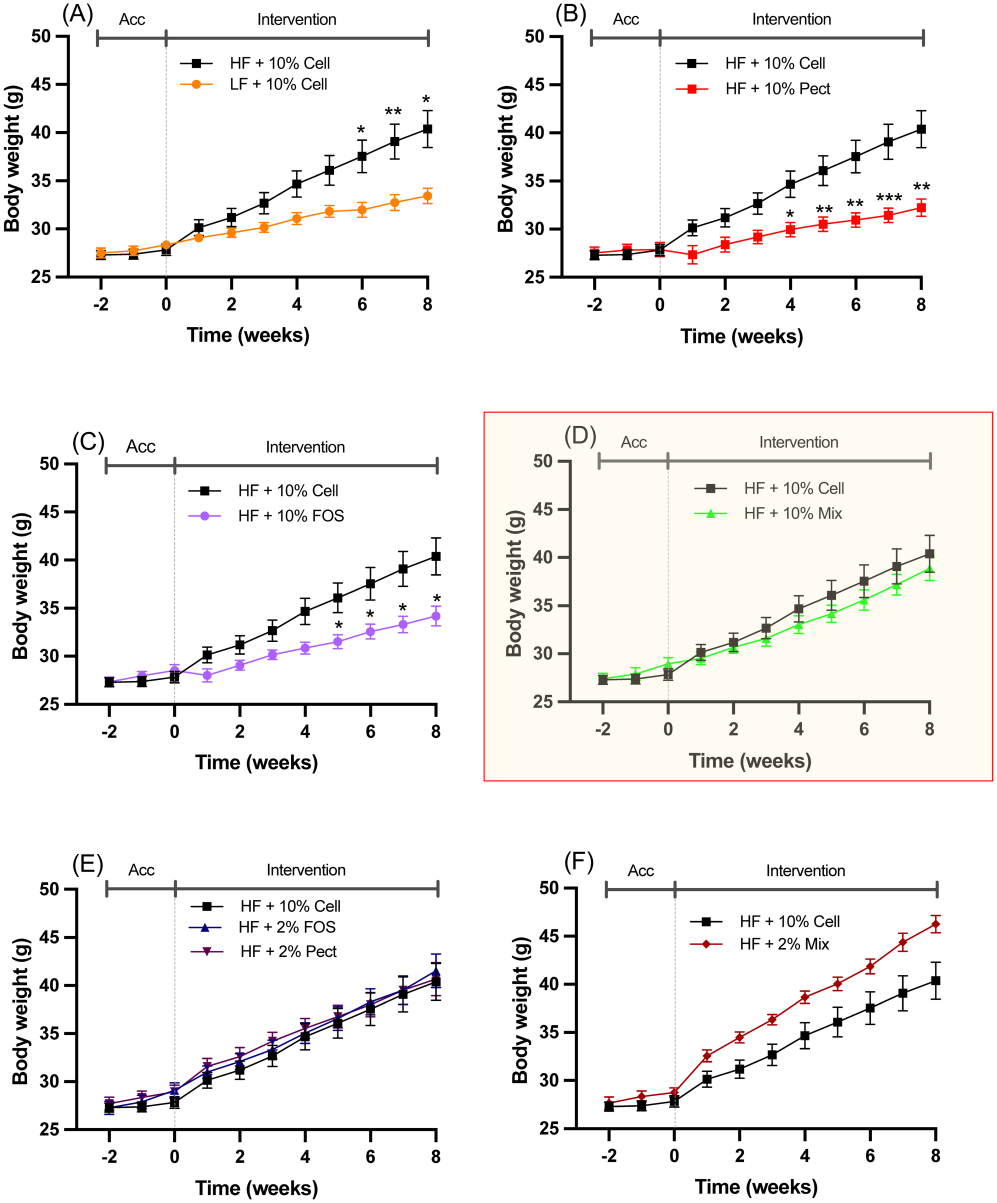
Body weights of mice fed different diets over the 2-week acclimation (Acc) and 8-week intervention period outlined in Figure 1. For clarity, the different dietary interventions relative to the effect of HF + 10% cellulose (HF + 10% Cell) are each shown on graphs **A-F**. **(A)**, LF + 10% Cell; **(B)**, HF + 10% Pect; **(C)**, HF + 10% FOS; **(D)**, HF + 10% Mix; **(E)**, HF + 2%FOS and HF + 2% Pect; **(F)**, HF + 2% Mix. Data show mean ± SEM, *n* = 8. Data for all treatments were analyzed by one-way ANOVA at each time point followed by Tukey’s multiple comparison for statistical significance. * (*p* < 0.05) and ** (*p* < 0.01) show statistically significant differences between interventions at the same time point on each graph. Graph **D** is highlighted to emphasize the lack of response to 10% mixed fiber relative to the suppressive effects of either 10% pectin (Pect) **(B)** or 10% FOS **(C)**.

Mice fed a HF diet +10% cellulose over an 8-week period (weeks 2–10) gained significantly more body weight than mice fed a LF diet (Figure 2A). In mice fed HF + 10% Pectin or HF + 10% FOS, body weight was significantly suppressed by each treatment relative to HF + 10% cellulose (Figures 2B,C). By contrast, mice fed HF + 10% mix fibers for 8 weeks, showed no significant difference in body weight relative to HF + 10% cellulose (Figure 2D). Similarly, mice fed HF + 2% pectin or 2% FOS were not significantly different in body weight from HF + 10% cellulose fed mice at any time point (Figure 2E). Mice fed HF + 2% mix showed an apparent, but statistically non-significant increase, in body weight relative to the HF + 10% cellulose fed mice (Figure 2F). There was no significant variation in body weight during the initial 2-week acclimation period (week 0–2) in any of the treatment groups.

### 3.2 Effects of fiber on adiposity

**Figure 3:**
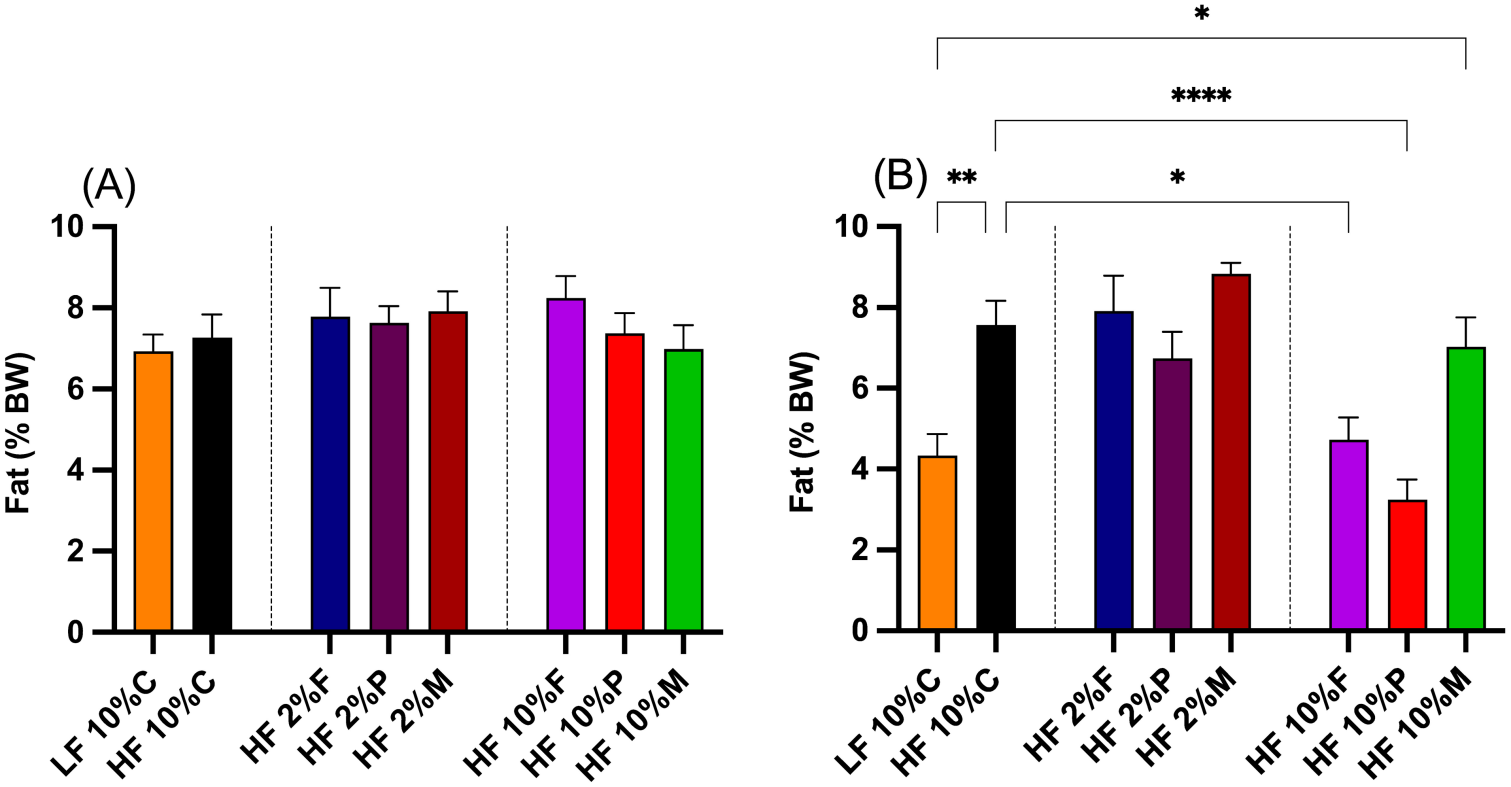
Adiposity of mice at the beginning **(A)** and the end **(B)** of the intervention period. Fat mass at the start of the intervention period was measured by Echo MRI, while the fat mass at the end of the intervention period was based on dissected epididymal and retroperitoneal fat pad weights measured at week 8. Data show mean ± SEM, *n* = 8. Data were analyzed by one-way ANOVA followed by Sidak’s multiple comparison test for statistical differences. * (*p* < 0.05), ** (*p* < 0.01) and **** (*p* < 0.0001) show statistically significant differences between treatments for indicated comparisons. LF, low fat; HF, high fat; C, cellulose; F, fructooligosaccharide; P, pectin; M, mixed fiber.

The effects of fiber on body adiposity were measured from the amount of fat expressed as a percentage of overall body weight at the start and end of the study (Figures 3A,B). At the start of the experiment there were no significant differences in percentage body fat between the mice on any of the treatments (Figure 3A). After 8 weeks of treatment (weeks 2–10), the percentage fat of mice fed HF + 10% cellulose significantly increased to almost double the levels of the LF control mice (Figure 3B). Those mice fed HF + 10% pectin or 10% FOS showed significantly reduced levels of percentage body fat relative to HF + 10% cellulose fed mice, with levels similar to those of the LF control fed mice (Figure 3B). Mice fed the HF + 10% mix diet, showed a significant increase in percentage fat mass relative to LF controls, but no significant difference relative to HF + 10% cellulose fed mice (Figure 3B). For all mice fed 2% fibers (pectin, FOS or MIX) no significant differences relative to the HF + 10% cellulose fed mice were observed (Figure 3B).

### 3.3 Effects of fiber on cecal fermentation acids

**Figure 4:**
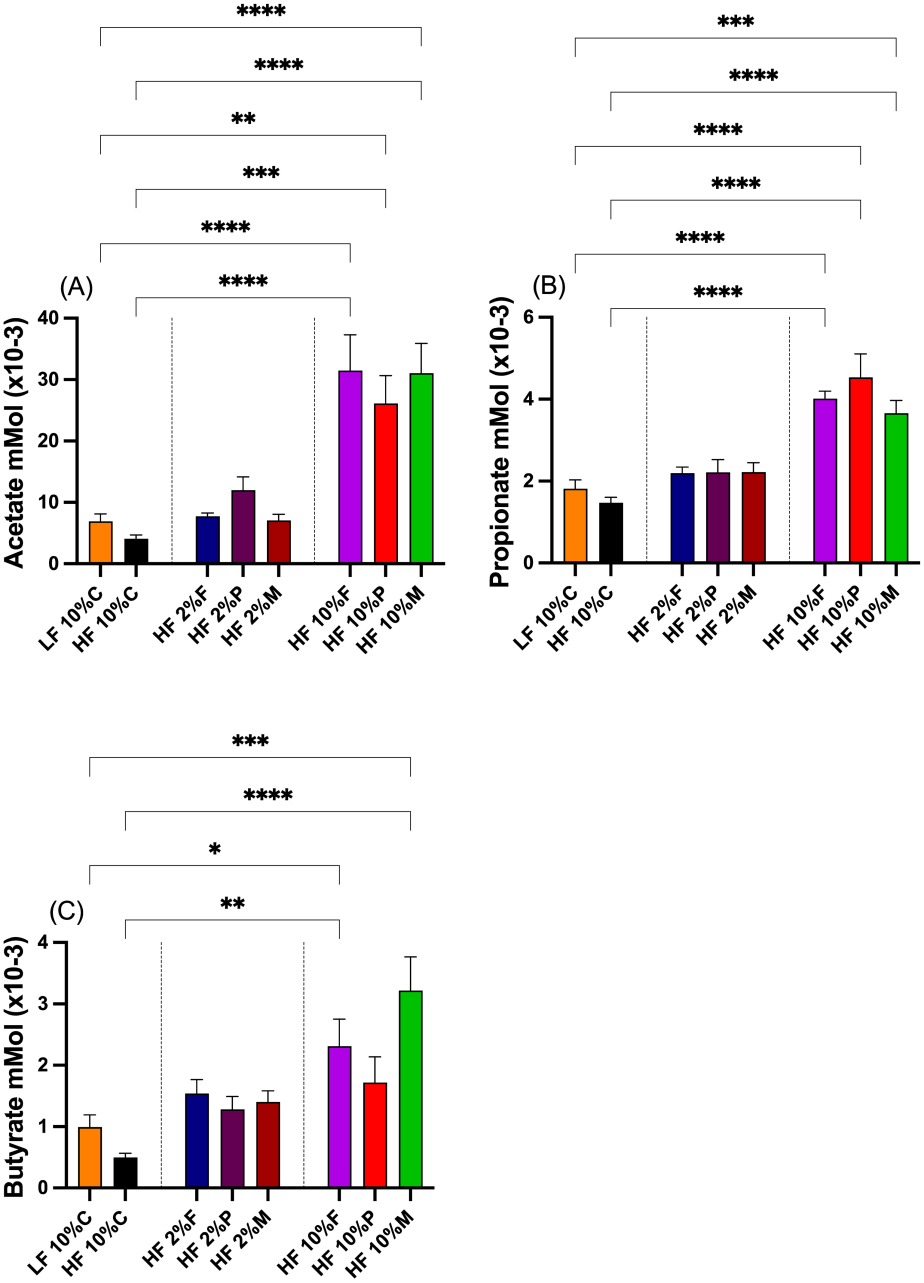
Total short chain fatty acid levels, **(A)**, acetate, **(B)** propionate and **(C)** butyrate, in the ceca of mice on different dietary treatments. Data show mean ± SEM, *n* = 8. Data were analyzed by one-way ANOVA followed by Sidak’s multiple comparison test for statistical differences. *(*p* < 0.05), **(*p* < 0.01), ***(*p* < 0.001) and ****(*p* < 0.0001) show statistically significant differences between treatments for indicated comparisons. LF, low fat; HF, high fat; C, cellulose; F, fructooligosaccharide; P, pectin; M, mixed fiber.

The effects of fiber on the total SCFA levels, acetate, butyrate and propionate, are shown in Figures 4A–C. Inclusion of 10% pectin, FOS or Mix significantly elevated total acetate levels in the cecum relative to both the LF control and HF + 10% cellulose fed mice. At the 2% dose neither pectin, Fos nor Mix were significantly altered relative to either the LF control or the mice fed HF + 10% cellulose (Figure 4A). A similar pattern of response was seen for propionate (Figure 4B) with only mice fed 10% pectin, FOS or Mix showing elevated propionate relative to either the LF control or the HF + 10% cellulose treatments. For butyrate while both 10% FOS and Mix treatments significantly elevated butyrate levels above both the LF control and the HF + 10% cellulose treatments, 10% pectin failed to significantly elevate butyrate above the LF and HF controls (Figure 3C). None of the 2% fiber treatments raised butyrate above these controls.

### 3.4 PYY and GLP-1 responses to dietary fiber

**Figure 5:**
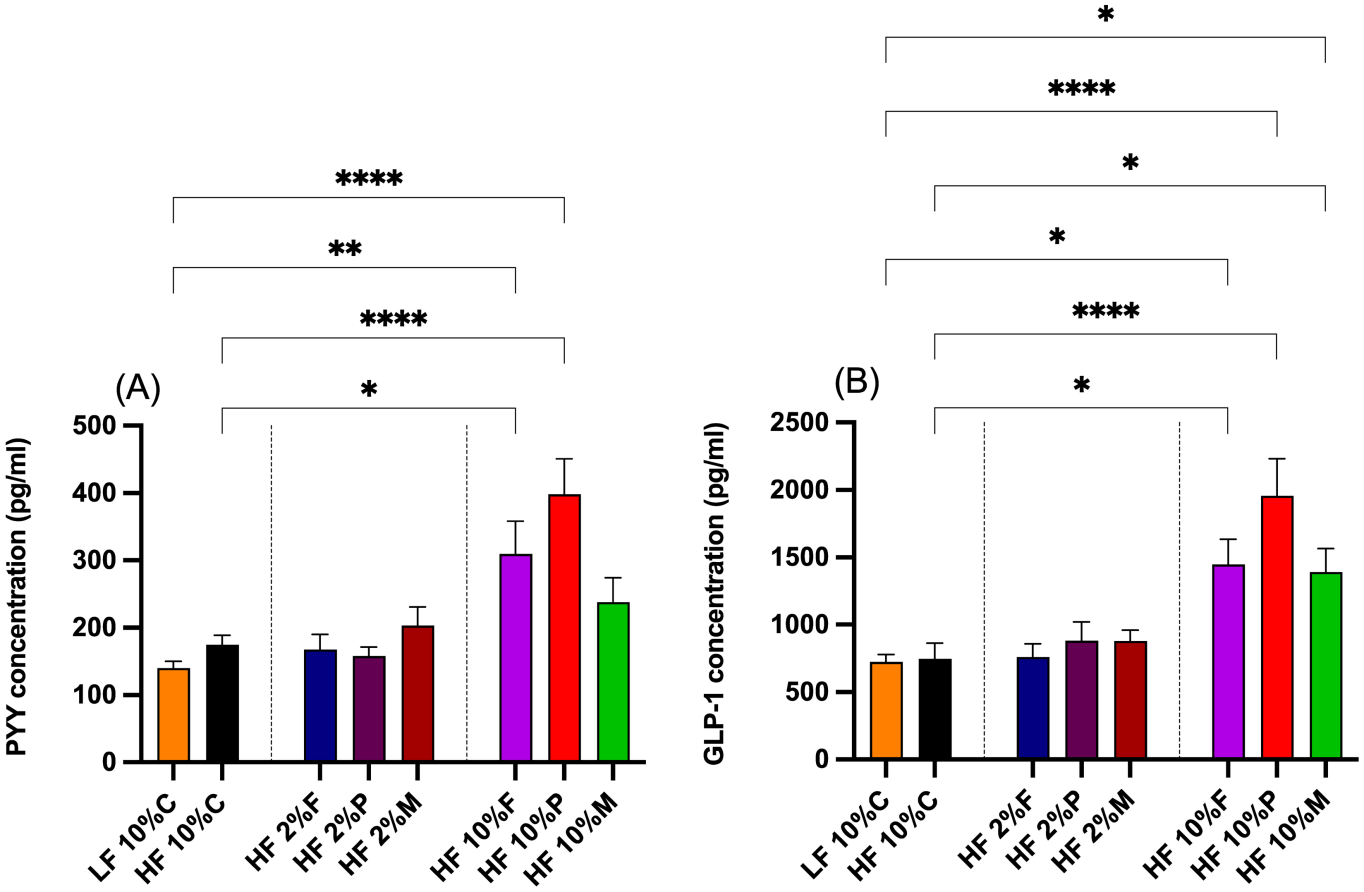
Plasma levels of gut hormone PYY **(A)** and GLP-1 **(B)** taken at the end of the experiment at week 8. Data show mean ± SEM, n = 8. Data were analyzed by one-way ANOVA followed by Sidak’s multiple comparison test for statistical differences. *(*p* < 0.05), **(*p* < 0.01), ***(*p* < 0.001) and ****(*p* < 0.0001) show statistically significant differences between treatments for indicated comparisons. LF, low fat; HF, high fat; C, cellulose; F, fructooligosaccharide; P, pectin; M, mixed fiber.

The gut hormone responses to dietary fiber treatments are shown in Figures 5A,B. Both 10% pectin and 10% FOS fibers stimulated significant increases in plasma PYY levels above both the LF and the HF + 10% cellulose controls. By contrast neither the 10% Mix fiber treatment nor any of the 2% fibers treatments significantly raised plasma PYY concentrations above the control levels (Figure 5A).

In the case of GLP-1 all the 10% fiber treatments (pectin, FOS and Mix) increased the plasma levels above both the LF and HF controls, while the 2% fiber treatments were without significant effect (Figure 5B).

For PYY and GLP-1 there appeared to be a graded effect where pectin had a stronger effect than FOS, which in turn was stronger than for the mixed fibers.

### 3.5 Effects of fiber on gut microbiota

The role of gut microbiota in the weight loss response to fiber was assessed using 16S rRNA gene sequencing of microbial DNA isolated from cecal microbiota and subsequent analysis using Mothur software. For analysis, the LF group was used as the control against which all the fiber treatments, pectin, FOS and Mix were compared.

**Figure 6:**
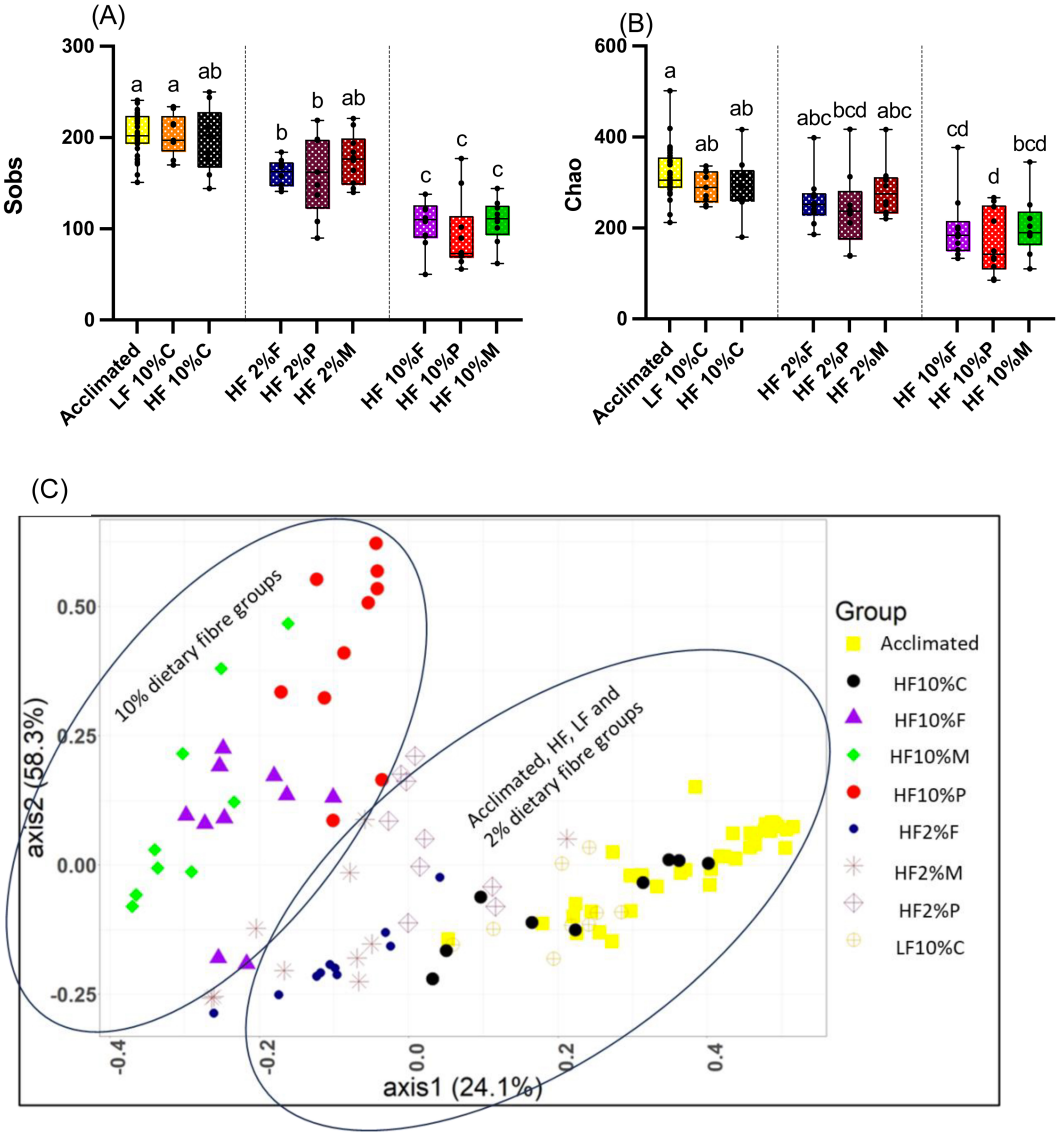
Alpha diversity measurements for cecal samples relative to treatment. Box and whisker plots show observed species richness (sobs) **(A)** and chao estimated species richness **(B)**. The horizontal line in the box represents the median. Data were analyzed by one-way ANOVA with Tukey’s *post hoc* test. Letters above individual treatments, which are not the same, indicate statistically significant differences (*p* < 0.05). **(C)** show shows Bray Curtis beta diversity (PCoA plot) clustering patterns between cecal microbiota samples and the different dietary treatments. The clustering patterns were confirmed as being statistically significantly different using the AMOVA (analysis of molecular variance) function implemented in Mothur. LF, low fat; HF, high fat; C, cellulose; F, fructooligosaccharide; P, pectin; M, mixed fiber.

Illumina MiSeq sequencing showed that dietary fiber strongly influenced the composition of the gut microbiota. The highest alpha diversities (sobs and chao) were observed in the cecal microbiota of mice during acclimation and in the LF and HF + 10% cellulose control groups, while the lowest were observed in mice fed each of the 10% fiber groups (Pectin, FOS and Mix). Mice given the 2% fibers showed intermediate diversity (Figures 6A,B). The pattern of response was similar between the observed richness (sobs) and estimated richness (chao) (Figures 6A,B).

Given that significant differences in alpha diversity between the cecal microbiota of mice in the cellulose control group and the soluble dietary fiber groups were observed, further analysis was undertaken to assess beta diversity which have been visualized using Bray Curtis-based principal coordinate plots (Figure 6C). Nonparametric analysis of molecular variance (AMOVA) showed significant differences in clustering, which were treatment-dependent (*p* < 0.001). Notably all the 10% fiber treatments (pectin, FOS and Mix) were distinct from the LF and HF controls and each fiber group was distinct from the other (*p* < 0.001; Figure 6C).

**Figure 7:**
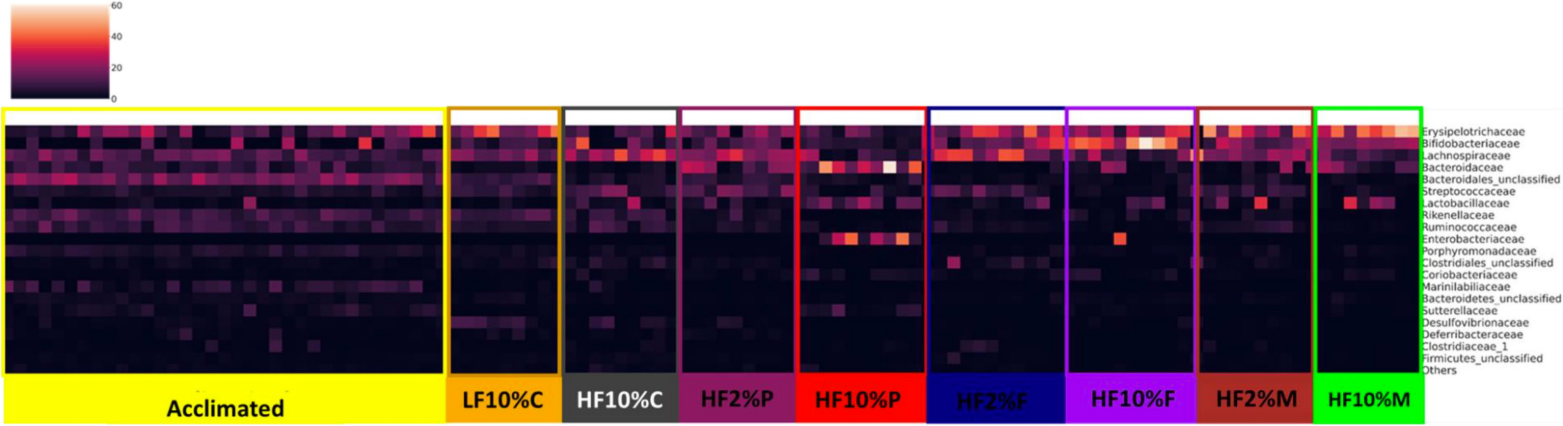
Heatmap showing bacterial proportional abundance at the Family level relative to the different dietary treatments as well as the acclimation period. Color intensities represent proportional abundance at the family level of classification with lighter colors representing higher proportional abundance and darker colors representing lower proportional abundance. LF, low fat; HF, high fat; C, cellulose; F, fructooligosaccharide; P, pectin; M, mixed fiber.

With a clear effect of dietary fiber on gut microbiota composition revealed, we next examined whether specific bacterial taxa were associated with the distinct dietary treatments. Figure 7 shows a heat map of microbiota abundance at the family level. Distinct patterns of expression can be seen for each treatment, which can be compared not only against the LF and HF + 10% cellulose controls but also against the pre-experimental (acclimation) profile. In response to the HF + cellulose diet, there is an increased abundance of *Lachnospiracae* (*p* < 0.05) but reduced abundance of *Erysipelotrichacae* (*p* < 0.05) relative to the LF and acclimation profiles. While HF + 2% pectin is like the HF control, HF + 10% pectin shows a major shift in profile with notable increases in abundance of *Bacterioidaceae*, *Lactobacillaceae* and *Enterobacteriaceae* (*p* < 0.001; Figure 7). For HF + FOS treated mice, there is also a notable increase in abundance of *Erysipelotrichacae* in both the 2 and 10% FOS fed mice relative to the HF control to levels similar to those of the LF control. Aside from *Erysipelotrichacae*, the profile of the 2% FOS fed mice are like the HF control, whereas there is a notable increase in *Bifidobacteriaceae* in the 10% FOS fed mice. In the HF + 2 and 10% Mix fed mice, there is an increased abundance of *Erysipelotrichacae* relative to the HF control, which appears to be greater than the LF control. There is also an increased abundance of *Bifidobacteriaceae* in the HF + 2 and 10% Mix fed mice relative to the HF control, although this is notably smaller than seen in the 10% FOS treated mice.

**Figure 8:**
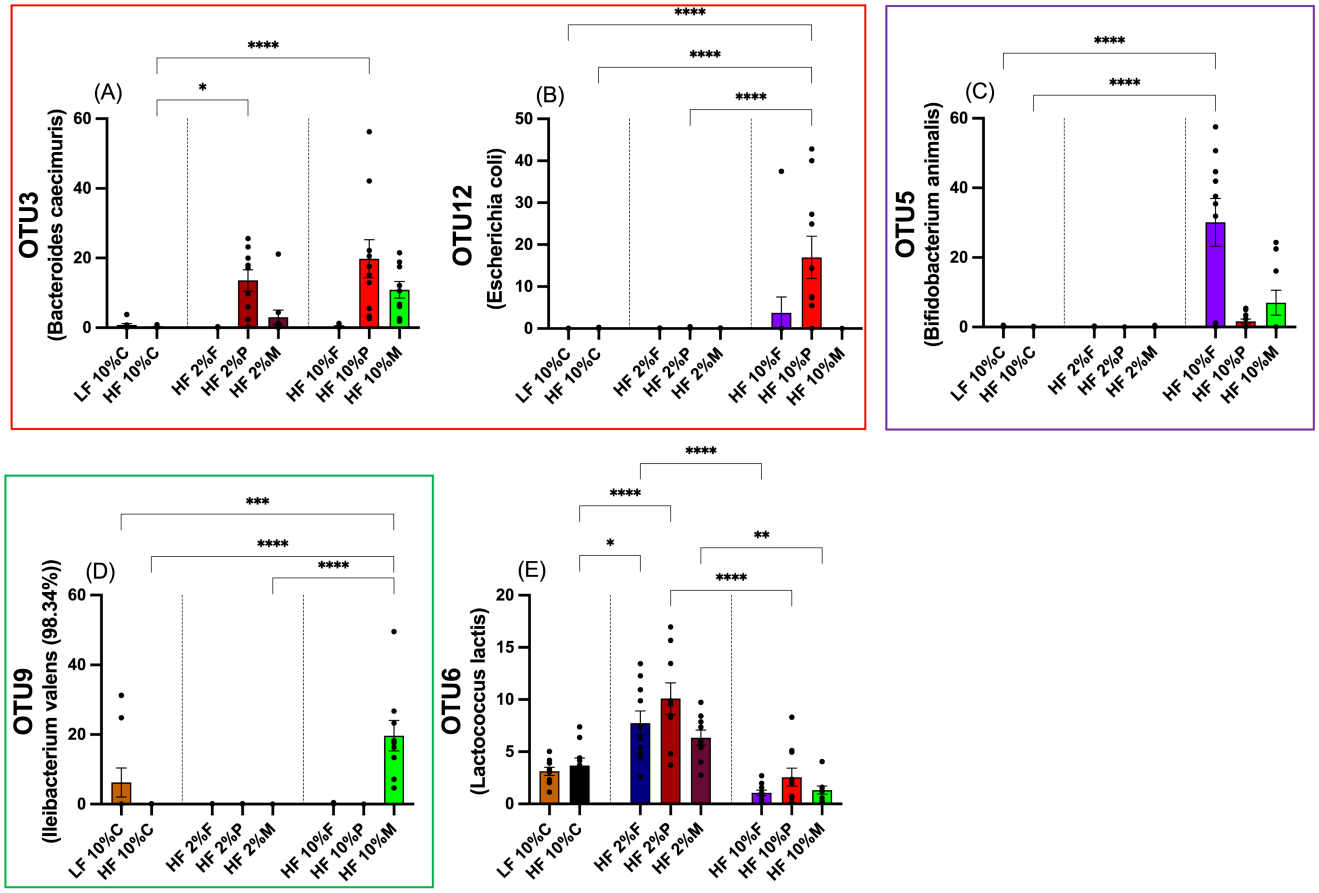
Percentage proportionally abundance of selected OTUs, which highlights the distinct responses to the different fiber treatments. Data show mean ± SEM, *n* = 8. * (*p* < 0.05), ** (*p* < 0.01) and ***(*p* < 0.001) show statistically significant differences between treatments for indicated comparisons: **(A)** OTU3; **(B)** OTU12; **(C)** OTU5; **(D)** OTU9 and **(E)** OTU6. Data were analyzed by one-way ANOVA and statistically significant differences between treatments were determined by using Metastats in Mothur and the Benjamini-Hochberg correction. LF, low fat; HF, high fat; C, cellulose; F, fructooligosaccharide; P, pectin; M, mixed fiber.

At the OTU level 10% Pectin stimulates increased abundance of *Bacteriodes caecimuris* (OTU3) and an increased abundance of *Escherichia coli* (OTU12) (*p* < 0.001; Figures 8A,B). While 2% pectin also elevates *B. caecimuris* (OTU3) (*p* < 0.01; Figure 8A), the magnitude of this increase is less than for 10% pectin and there is no effect on *E. coli* (OTU12) (Figure 8B). The main effect of 10% FOS is increased abundance of *Bifidobacterium animalis* (OTU5) (*p* < 0.01) and although there appears to be a small increase in *B.animalis* in response to 10% Mix, this is not significantly elevated relative to the HF control (Figure 8C). 10% Mix fibers significantly increased the abundance of *Ileibacterium valens* (OTU9) (*p* < 0.001; Figure 8D).

OTU6 (*Lactococus lactis*) is an example of a bacterium showing increased abundance when the diets were supplemented with low doses of fiber but reduced abundance in the presence of high doses of fiber (10%) (Figure 8E). The full range of responses of OTUs 1–12 are shown in Supplementary Figure S1. These data also show how the bacteria changed in abundance relative to the pre-experimental acclimation period. Of note is the suppression of *Alistipes putredinis* (OTU8) by all dietary treatments relative to the acclimation period and *Alistipes montrealensis* (OTU10) by all the fiber treatments relative to the LF control and the acclimation period. For the remaining OTUs (1,2,4,7 & 11), there was no obvious pattern of response related to diet (Supplementary Figure S1).

### 3.6 Effects of fibers on gut epithelial gene expression by RNA seq analysis

RNA seq was used to assess gene expression in the colon epithelium of mice fed the different fiber diets.

Bioinformatic analysis showed that relative to the HF control, inclusion of dietary fiber in the diet increased the number of genes up-or down-regulated. However, the number of genes up and downregulated by the single fiber treatments (pectin and FOS) were vastly greater than those for the mixed fiber diet. 1,200 genes were upregulated in pectin, 1,261 genes by FOS and 113 for mixed fiber. 1,134 genes were down regulated by pectin, 1,580 genes by FOS and 58 by mixed fiber. The many gene expression changes were reflected in changes in multiple pathways, but it was difficult to discern a specific and characteristic response related each of the treatments, although it was noted that increased expression of immune-related gene was evident in the pectin and FOS treatments.

To try and focus the gene expression changes, the bioinformatic analysis was concentrated on those genes that were either up or down regulated by 10% pectin or FOS but not by 10% Mix diets. This was because we had observed strong effects of 10% pectin and 10% FOS in preventing HF diet induced weight gain, which was not observed when 10% mixed fibers were used (see Figure 2).

Of the 1,200 genes significantly up-regulated (*p* < 0.05) by 10% pectin relative to HF + 10% cellulose, this reduced to 119 when a filter of 2-fold change was applied. Likewise, for 10% FOS of the 1,261 genes significantly up-regulated (*p* < 0.05) relative to the HF control this reduced to 174 genes when a 2-fold cut-off was applied. For the 10% Mix the 113 upregulated genes relative to the HF control, was reduced to 48 genes at a 2-fold change threshold.

Table 1 shows the top 20 upregulated genes in terms of fold changes induced by 10% pectin and 10% FOS, but not significantly changed in expression in 10% Mix fed mice, as assessed by RNA sequence analysis. Although the rank order and magnitude of gene expression is slightly different between pectin and FOS, there is nonetheless a high degree of commonality in the top 20 gene expression changes. Many Ighv and Igkv genes represent some of the highest fold changes in response to both fibers, indicating some form of immune response to the diets. It should be noted that induction of Ighv and Igkv genes was not unique to pectin and FOS treatments, since large changes in genes such as Ighv1-77 and Igkv8-28 were seen in Mix as well as pectin and FOS treatments. Other notable genes induced by pectin and FOS include robust increases in Reg3β and *γ*, which are known antimicrobial proteins. Another upregulated gene in both pectin and FOS treatments is the gut hormone CCK, which is a known appetite suppressive hormone.

**Table 1:** Colon epithelial genes significantly upregulated by 10% Pectin and 10% FOS but not 10% Mix by RNA seq.

Gene	FC (Pect)	pAdj_PECT	Gene	FC (FOS)	pAdj_FOS
Reg3b	12.7796603	8.002E-05	**Ighv1-74**	23.4566858	0.00044676
**Reg3g**	12.2165749	4.7482E-05	**Igkv8-30**	18.1730449	1.1489E-05
**Ighv8-8**	9.66183506	0.0058368	**Ighv5-4**	12.5364484	0.00410988
**Ighv1-74**	9.04000261	0.00800412	**Ighv1-19**	11.4909921	0.00091526
**Igkv12-41**	8.98900185	0.01625287	**Ighv8-8**	8.94577728	0.01646543
**Ighv1-19**	8.93876725	0.00106107	**Igkv12-41**	8.92148231	0.03110282
**Igkv8-30**	6.46592852	0.00178526	**Igkv6-15**	7.81646337	0.00014604
**Akr1c18**	5.83959003	5.2841E-06	**Ighv1-80**	6.06663459	0.01982643
Acaa1b	5.29147005	1.1114E-08	**Igkv4-57-1**	5.44889518	0.04595082
**Ighv5-4**	5.07950021	0.03934688	**Reg3b**	5.17704615	0.02349748
**Igkv4-57-1**	4.9259577	0.02972981	**Reg3g**	4.72737001	0.02617924
**Ighv1-80**	4.36257145	0.02879206	**Akr1c18**	4.50459326	0.00103146
**Igkv6-15**	3.77116369	0.00610699	**Igkv19-93**	4.12282414	0.00816981
Slc27a2	3.73119422	7.5853E-07	**Cck**	4.07330373	1.4447E-05
St8sia5	3.59490636	7.3784E-08	**Prap1**	3.77115007	0.002712
Vnn1	3.53201029	3.1011E-07	Jchain	3.66281881	0.00212344
**Cck**	2.91997527	8.1403E-05	Igha	3.30341524	0.00571955
Gm32894	2.76299137	0.00098696	Igkc	3.23698858	0.00440199
**Prap1**	2.62950937	0.01158926	Reg3a	3.22472379	0.01291679
**Igkv19-93**	2.52603432	0.04357677	Cldn14	3.10840674	5.3787E-09

Bold indicates response seen in top 20 upregulated genes for both pectin and Fos. FC, fold change; pADj, *p* value adjusted for false discovery rate.

Table 2 shows the top 20 most down regulated genes in the colon epithelium by 10% pectin and 10% FOS treatments, but not significantly changed in expression in the 10% Mix fed mice. Again, the magnitude and rank order of expression was different between the two treatments, but at least 50% of the genes were common. A notable gene is leptin which was common to the pectin and FOS treatments as one of the most down regulated. As leptin is a marker of adiposity downregulation of this gene is consistent with the reduction in adiposity observed due to the treatments with pectin and FOS.

**Table 2:** Colon epithelial genes significantly downregulated by 10% Pectin and 10% FOS but not 10% Mix by RNA seq.

Gene	FC (Pect)	pAdj_PECT	Gene	FC (FOS)	pAdj_FOS
**Lep**	0.08704645	0.00025147	Slc5a7	0.05185803	0.00351771
**Trim67**	0.13916845	5.9845E-05	**Lep**	0.06421573	0.00632556
**Ctcflos**	0.14394844	0.00033446	**Adam12**	0.0954583	0.00128222
**Prr32**	0.14935426	0.00189643	**Nav3**	0.12237999	0.00620102
**Adam12**	0.16602672	3.1489E-05	**Trim67**	0.12633754	0.00806738
Mest	0.16654656	0.00026718	**Prr32**	0.12835976	0.02649011
**Npr3**	0.17492474	0.00132477	**Npr3**	0.12938884	0.01031379
**Aqp7**	0.1773088	0.00020122	**Aqp7**	0.13424881	0.00395845
**Plin4**	0.17927442	0.00065675	Kcna5	0.13659852	0.01545536
Slc7a10	0.18162703	0.0003134	**Plin4**	0.14055294	0.00758611
Sncg	0.19070974	0.00093459	Lhx6	0.14377443	0.01043843
**Nav3**	0.19426703	0.00047532	Ces2f	0.14449319	0.00143714
Cd36	0.20735154	0.00016225	Gm51702	0.14798936	0.0002049
Adamts5	0.21198867	0.00135012	Gm54079	0.15515236	0.00024373
Aox1	0.22124483	2.9793E-06	Gm40696	0.15780063	0.00400146
Gdf10	0.22320004	0.00100857	Fgf13	0.16083766	0.02163942
Aoc3	0.22506737	0.00215344	Sncg	0.16133681	0.01170483
Fgf13	0.22573715	0.00283352	**Ctcflos**	0.16756475	0.02696118
**Tshr**	0.22821581	0.00263066	**Tshr**	0.17450061	0.01477355
Meox2	0.23104069	0.00043521	Sfrp5	0.17504855	0.01061837

Bold indicates response seen in top 20 downregulated genes for both pectin and Fos. FC, fold change; pADj, *p* value adjusted for false discovery rate.

## 4 Discussion

This study confirms that dietary fiber can act as a powerful brake on adiposity and body weight gain in mice fed high fat diets. This effect was seen when single dietary fibers (pectin and FOS) were added to the diet at 10% w/w but not at a lower dose of 2%. The 10% dose was chosen since it has been used in previous rodent studies and shown to restrain body weight gain (Cani et al., 2006; Cani et al., 2004; Adam et al., 2015; Adam et al., 2014). Nonetheless, 10% (w/w) fiber is considered a high dose, being about double the recommended daily intake for American men (Adam et al., 2014; Turner and Lupton, 2011) and so 2% (w/w) fiber intake was chosen as a potentially more representative daily intake. A similar dose dependency has been observed for rodents in other studies, where a strong negative effect on body weight gain has been observed relative to a much weaker, albeit significant, suppressive effect at 3.3% pectin (Adam et al., 2015). In the present study, the effect was not replicated if the fiber content was maintained at 10% but was made up of 4 different fibers. This is an important finding as it indicates that for maximal effect, in terms of restraining body weight gain and adiposity, both fiber type and dosage must be optimal. In other words, in the 10% Mix diet, where each fiber was at 2.5%, the effects of the different fibers were not additive, but separate and distinct and more akin to using each fiber at a low dose alone. As the fibers used are all substrates for the gut microbiota, the data hint that at the lower doses the fibers were unable to stimulate sufficient shift in microbial profile to enable a negative effect on energy balance to occur. Whereas at the higher 10% dose with a single fiber (pectin and FOS) a more pronounced shift in microbiota profile occurred which is required for the restraint on body weight gain and adiposity.

The data from the 16S analysis of the microbiota indicate both at the family and OTU level that 10% pectin and 10% FOS stimulated distinct changes in the gut microbiota, which were not seen at the 2% doses. These patterns were also distinct from those seen in response to 10% Mix, as well as being distinct from each other. This implies that no single microbial profile explains the weight restraining effects of dietary fiber, rather they differ with fiber type and thus are fiber dependent.

FOS is a well-known substrate for the bifidobacteria and thus the major increase in the Bifidobacteriaceae at the family level and *B. animalis* (OTU5) in response to 10% FOS is consistent with this. B.animalis has been shown to have weight reducing effects when administered as a probiotic to mice fed a high fat diet (Stenman et al., 2014; Do et al., 2022; Niu et al., 2022). Thus, the induction of *B.animalis* (OTU5) by 10% FOS, without any significant changes by any of the other fiber treatments indicates that this is a specific microbiota response that could explain the body weight restraining response observed.

For Pectin (10%), the increase in Enterobacteriaceae and more specifically *E. coli* (OTU12) was a unique response. Additionally, pectin (10%) stimulated increased abundance of Bacteriodaceae, and specifically *Bacteriodes caecimuris* (OTU3), although this was also stimulated to a weaker extent by 2% pectin and 10% mix. It is unclear whether the weight restraining effects require both *E.coli* and *B.caecamuris*, or instead is associated with *E.coli* only. However, a recent study suggests that *E. coli* tends to aggravate, rather than ameliorate obesity in mice fed a HFD (Ju et al., 2023), implying that the main weight reducing effect may require *B. caecimuris*, rather than being associated only with *E.coli*. While no association between *B. caecimuris* and body weight or adiposity has been reported to date, it is interesting that Bacteriodes is reported to increase in patients who had undergone bariatric surgery and associated weight loss (Kim et al., 2022).

10% Mix had no effect on body weight gain and adiposity, yet it did yield a unique microbial response: namely a substantial increase in abundance of *Ileibacterium valens*, a member of the Erysipelotrichacae family, which was not seen in response to any of the other dietary treatments. Increased abundance of *Ileibacterium valens* has been associated with weight loss in high fat high sucrose fed obese mice supplemented with *trans*-10,*cis*-12 Conjugated Linoleic Acid (t10,c12-CLA) (Den Hartigh et al., 2018). In this study, the increased abundance of *I. valens* was insufficient either alone or in magnitude to elicit a weight restraining response.

From the above, it is evident that the microbial profiles for the pectin, FOS and Mix responses are quite different, whereas the level of the fermentation acids, acetate, butyrate, and propionate, the responses are remarkably similar between all the fibers, except for a lack of effect of pectin on butyrate. As the 10% FOS and 10% MIX responses were almost identical for each of the SCFAs, yet the weight-restraining responses were different, it seems unlikely that the fermentation acids are part of the primary mechanism involved in the weight regulatory response. Furthermore, since all fiber treatments stimulated robust increases in acetate, this implies that a direct effect of acetate on appetite centers in the hypothalamus (Anastasovska et al., 2012; Frost et al., 2014) are unlikely to explain the differential weight-restraining responses observed.

In terms of gut hormones, the pattern of plasma PYY levels correlated with the fiber treatments, with significantly elevated PYY in response to 10% pectin and 10% FOS, but for 10% Mix PYY was not significantly increased above the LF or HF controls. By contrast GLP-1 was elevated in response to all the 10% fiber treatments. These results imply that either plasma PYY has a primary role in the anorexigenic responses to the fibers or that elevation in both GLP-1 and PYY are required to achieve suppression of body weight. The data also imply that microbial regulation of GLP-1 and PYY synthesis and/or release can be controlled by independent mechanisms. Given that the SCFAs, acetate, propionate and butyrate, increased in response to all fiber treatments, then the data imply that PYY synthesis and/or release are regulated by an SCFA-independent pathway.

In the search for additional or alternative mechanisms to control food intake and body weight, we used RNA seq of the gut epithelial tissue. This revealed several other potential regulators of energy homeostasis that could contribute to the fiber responses observed. This includes the Reg3 family of proteins (Shin et al., 2023). Reg3 proteins are antimicrobial peptides, produced mainly in the intestinal Paneth cells, from where they enter the gut lumen to exert bacteriocidal activity. The family of proteins include four variants in mice (Reg3α,*β*,*δ* and *γ* and two variants in humans, Reg3α and γ). It has been reported elsewhere that dietary fiber, increases Reg3γ in mice (Paone et al., 2022; Zou et al., 2018) and the SCFA propionate, increases Reg3 expression in murine small intestinal organoids (Bajic et al., 2020).

Similarly for CCK, a well-known anorexigenic gut hormone produced by the gut I-cells (Cawthon and de La Serre, 2021), it was found that by RNA seq, CCK gene expression was significantly elevated by 10% pectin and 10% FOS but not by 10% Mix. These RNA seq data suggest that fibers may have differential effects via Reg3 proteins and CCK, which may underpin the differential body weight response observed.

In this study, we show the powerful counteractive effect that dietary fiber can have on high fat diet induced weight gain, substantiating findings from previous studies (Anastasovska et al., 2012, Cani et al., 2006, Adam et al., 2016). However, we also show that both the dose and the nature of the fiber is critically important to the efficacy of the response. At 2% dose, neither pectin nor FOS were effective at restraining HF induce weight gain, yet at a 10% dose they were. Importantly however, fiber was only effective when administered as a single fiber type, since 10% fiber content, made up of 4 different fibers (2.5% each) were without weight restraining effect. Given the difference in the microbiota profiles shown to each of the fiber treatments, it seems that the single 10% fiber dose was required to push the microbiota response to an individual fiber above a threshold abundance. In the 10% Mix, the microbiota response was ‘effectively diluted’.

It is important to note that the diversity of microbiota was reduced in response to each of the 10% fiber treatments, relative to the LF and HF controls as well as the 2% fiber treatments. This may be an inevitable consequence of the use of high dose (10%) single or small mix of dietary fibers, which stimulate the growth of specific gut the microbiota, but it also raises the question of whether this is a healthy outcome, particularly if maintained over the long term. Reduced microbial diversity or dysbiosis may upset the homeostatic balance of benign or beneficial and pathogenic bacteria, potentially allowing overgrowth of the less desirable bacteria, which not only could have negative health consequences, but also may minimize the long-term beneficial effect of the elevated fiber. Dysbiosis has been implicated in a wide range of diseases including inflammatory bowel disease (IBD), obesity, allergic disorders, type 1 diabetes mellitus, autism, obesity, and colorectal cancer (CRC) (DeGruttola et al., 2016). Nonetheless, a recent large-scale study of over 21,000 individuals has shown that omnivores have greater microbiome diversity than either vegans or vegetarians, and yet due to meat consumption the omnivore diets are associated with the more negative cardio-metabolic health outcomes (Fackelmann et al., 2025). Thus, the relationship between gut microbial diversity and health is clearly more complex than just lower diversity means less healthy. In this regard, the gut epithelium showed some major shifts in immune related gene expression in response to high dose fiber treatment. Whether these changes are positive or negative and what the long-term consequences of a low diversity gut microbiota has on health requires further investigation and understanding.

## 5 Conclusion

In summary, this study shows that high dose single fibers (pectin and FOS), but not a mixture of fibers of equivalent dose, are highly effective in restraining weight and adipose tissue gain in mice fed a high fat diet. The study also shows for the first time that each fiber treatment stimulates a distinct gut microbiota response, which seems likely to be key to efficacy. The study also suggests that PYY has a role in the weight restraining response to dietary fiber with other factors such Reg3 protein and CCK potentially also having a role.

## Data Availability

The data discussed in this publication have been deposited in NCBI’s Gene Expression Omnibus (GEO) and are accessible through GEO Series accession number GSE288466 (https://www.ncbi.nlm.nih.gov/geo/query/acc.cgi?acc=GSE288466).
