# Supplementary material for: Single, but not mixed dietary fibers suppress body weight gain and adiposity in high fat-fed mice
Source: Front Microbiol. 2025 Feb 12;16:1544433. doi: 10.3389/fmicb.2025.1544433 (PMC11861375; doi:10.3389/fmicb.2025.1544433)
Supplement: Supplementary file 1 [file Data_Sheet_1.pdf]

Supplementary data to:

Single, but not mixed dietary fibers suppress body weight gain and adiposity in high fat-fed mice.

Swang M. Shallangwa[1], Alexander W. Ross[1], Peter J. Morgan[1*]

Table S1: Diet compositions used

	LF 10% Cell		HF 10% Cell		HF 10% Pectin		HF 2% Pectin		HF 10% FOS		HF 2% FOS		HF 10% Mixed		HF 2% Mixed	
%	gm	kcal	gm	kcal	gm	kcal	gm	kcal	gm	kcal	gm	kcal	gm	kcal	gm	kcal
Protein	18	20	23	20	24	20	23	20	24	20	23	20	24	20	23	20
Carbohydrate	64	70	40	35	36	30	39	34	36	30	39	34	36	30	39	34
Fat	4	10	23	45	24	45	23	45	24	45	23	45	24	45	23	45
Total		100		100		95		99		95		99		95		99
kcal/gm	3.6		4.5		4.7		4.6		4.8		4.6		4.7		4.6	
**Ingredient**	**gm**	**kcal**	**gm**	**kcal**	**gm**	**kcal**	**gm**	**kcal**	**gm**	**kcal**	**gm**	**kcal**	**gm**	**kcal**	**gm**	**kcal**
Casein	200	800	200	800	200	800	200	800	200	800	200	800	200	800	200	800
L-Cystine	3	12	3	12	3	12	3	12	3	12	3	12	3	12	3	12
Corn Starch	452.2	1809	72.8	291	25.5	102	63.2	253	22.5	90	62.5	250	22.2	89	62.4	250
Maltodextrin 10	75	300	100	400	100	400	100	400	100	400	100	400	100	400	100	400
Sucrose	172.8	691	172.8	691	172.8	691	172.8	691	172.8	691	172.8	691	172.8	691	172.8	691
Cellulose, BW200	112	0	90	0	0	0	71	0	0	0	71	0	0	0	71	0
Soybean Oil	25	225	25	225	25	225	25	225	25	225	25	225	25	225	25	225
Lard	20	180	177.5	1598	177.5	1598	177.5	1598	177.5	1598	177.5	1598	177.5	1598	177.5	1598
Mineral Mix S10026	10	0	10	0	10	0	10	0	10	0	10	0	10	0	10	0
DiCalcium Phosphate	13	0	13	0	13	0	13	0	13	0	13	0	13	0	13	0
Calcium Carbonate	5.5	0	5.5	0	5.5	0	5.5	0	5.5	0	5.5	0	5.5	0	5.5	0
Potassium Citrate, 1 H2O	16.5	0	16.5	0	16.5	0	16.5	0	16.5	0	16.5	0	16.5	0	16.5	0
Vitamin Mix V10001	10	40	10	40	10	40	10	40	10	40	10	40	10	40	10	40
Choline Bitartrate	2	0	2	0	2	0	2	0	2	0	2	0	2	0	2	0
Pectin (Sigma 93854, 90% pure)	0	0	0	0	95	190	19.8	40	0	0	0	0	24	48	5	10
FOS (Orafti P95, 89% pure)	0	0	0	0	0	0	0	0	96.37	202	20	42	0	0	0	0
Synergy 1 (87% pure)	0	0	0	0	0	0	0	0	0	0	0	0	49.1	105	10.3	22
Beta-glucan (83.59% pure)	0	0	0	0	0	0	0	0	0	0	0	0	26	50	5.3	10
FD&C Yellow Dye #5	0.04	0	0	0	0	0	0.05	0	0.025	0	0	0	0.04	0	0	0
FD&C Red Dye #40	0.01	0	0.05	0	0	0	0	0	0	0	0.025	0	0	0	0	0
FD&C Blue Dye #1	0	0	0	0	0.05	0	0	0	0.025	0	0.025	0	0.01	0	0	0
**Total**	**1117.05**	**4057**	**898.15**	**4057**	**855.85**	**4058**	**889.35**	**4058**	**854.22**	**4058**	**888.85**	**4058**	**856.65**	**4058**	**889.3**	**4058**
**Pectin (%)**	**0.00**		**0.00**		**9.99**		**2.00**		**0.00**		**0.00**		**2.52**		**0.51**	
**FOS (%)**	**0.00**		**0.00**		**0.00**		**0.00**		**10.04**		**2.00**		**0.00**		**0.00**	
**Synergy 1(%)**	**0.00**		**0.00**		**0.00**		**0.00**		**0.00**		**0.00**		**4.99**		**1.01**	
**Beta-glucan (%)**	**0.00**		**0.00**		**0.00**		**0.00**		**0.00**		**0.00**		**2.54**		**0.50**	
**Cellulose (%)**	**10.03**		**10.02**		**0.00**		**7.98**		**0.00**		**7.99**		**0.00**		**7.98**	
**Fiber (%)**	**10.03**		**10.02**		**9.99**		**9.99**		**10.04**		**9.99**		**10.04**		**10.00**	

Supplementary Figure 1S

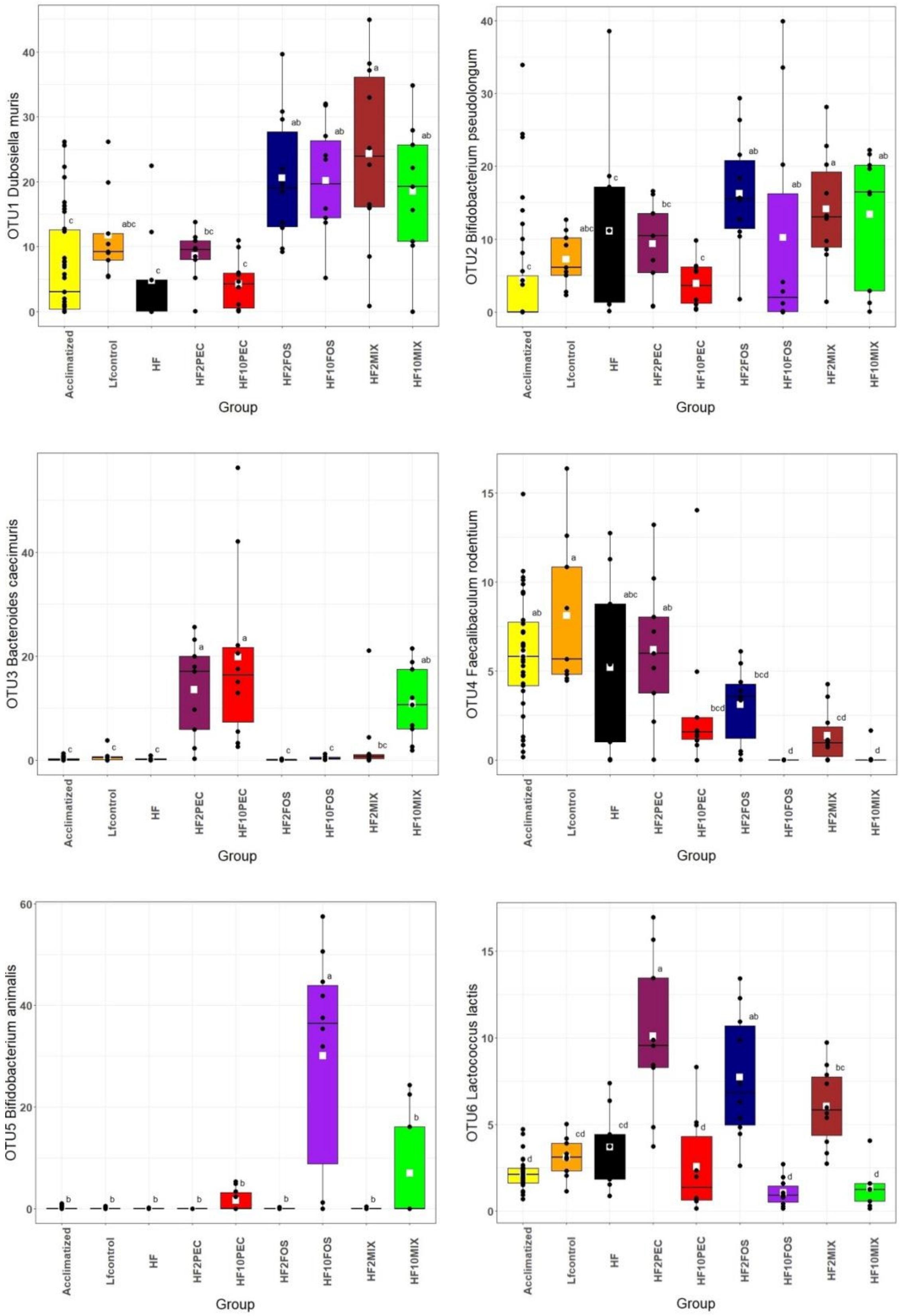

Supplementary Figure 1S contd

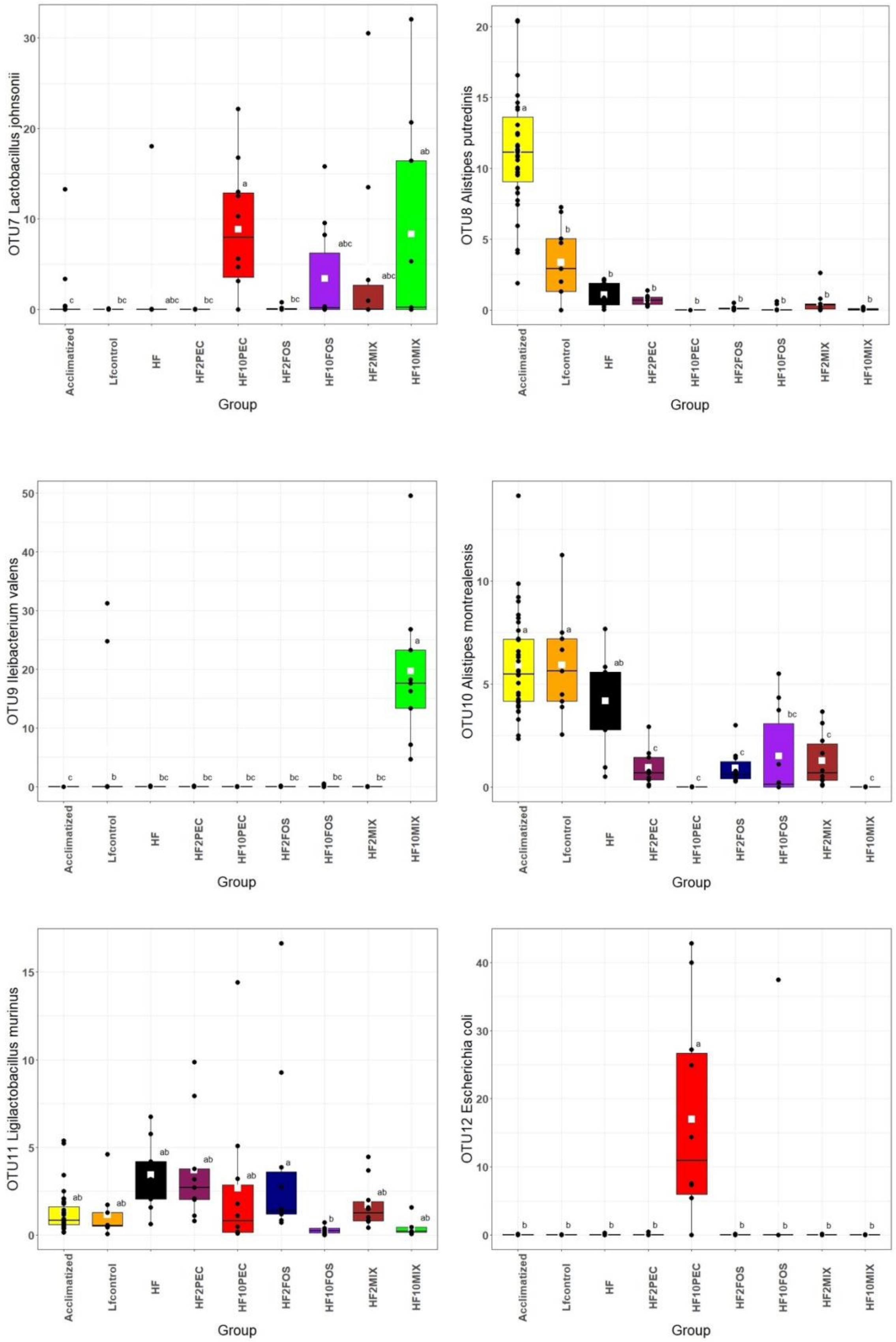

Figure 1S. Percentage proportionally abundance OTUs 1-12, showing the responses to the different fiber treatments. Data show mean ± SEM, n=8. Data were analyzed by one-way ANOVA and statistically significant differences between treatments were determined by using Metastats in Mothur and the Benjamini-Hochberg correction. Letters above individual treatments, which are not the same, indicate statistically significant differences ($p<0.05$)
